# Costs and outcomes of advance care planning and end-of-life care for older adults with end-stage kidney disease: A person-centred decision analysis

**DOI:** 10.1371/journal.pone.0217787

**Published:** 2019-05-31

**Authors:** Marcus Sellars, Josephine M. Clayton, Karen M. Detering, Allison Tong, David Power, Rachael L. Morton

**Affiliations:** 1 Kolling Institute, Northern Clinical School, Faculty of Medicine, The University of Sydney, Sydney, Australia; 2 Advance Care Planning Australia, Austin Health, Melbourne, Australia; 3 HammondCare Centre for Learning & Research in Palliative Care, Greenwich Hospital, Sydney, Australia; 4 Faculty of Medicine, Dentistry and Health Sciences, Melbourne University, Parkville, Victoria, Australia; 5 Sydney School of Public Health, The University of Sydney, Sydney, Australia; 6 Centre for Kidney Research, The Children’s Hospital at Westmead, Sydney, Australia; 7 Department of Nephrology, Austin Health, Melbourne, Australia; 8 NHMRC Clinical Trials Centre, The University of Sydney, Sydney, Australia; University of Mississippi Medical Center, UNITED STATES

## Abstract

**Background:**

Economic evaluations of advance care planning (ACP) in people with chronic kidney disease are scarce. However, past studies suggest ACP may reduce healthcare costs in other settings. We aimed to examine hospital costs and outcomes of a nurse-led ACP intervention compared with usual care in the last 12 months of life for older people with end-stage kidney disease managed with haemodialysis.

**Methods:**

We simulated the natural history of decedents on dialysis, using hospital data, and modelled the effect of nurse-led ACP on end-of-life care. Outcomes were assessed in terms of patients’ end-of-life treatment preferences being met or not, and costs included all hospital-based care. Model inputs were obtained from a prospective ACP cohort study among dialysis patients; renal registries and the published literature. Cost-effectiveness of ACP was assessed by calculating an incremental cost-effectiveness ratio (ICER), expressed in dollars per additional case of end-of-life preferences being met. Robustness of model results was tested through sensitivity analyses.

**Results:**

The mean cost of ACP was AUD$519 per patient. The mean hospital costs of care in last 12 months of life were $100,579 for those who received ACP versus $87,282 for those who did not. The proportion of patients in the model who received end-of-life care according to their preferences was higher in the ACP group compared with usual care (68% vs. 24%). The incremental cost per additional case of end-of-life preferences being met was $28,421. The greatest influence on the cost-effectiveness of ACP was the probability of dying in hospital following dialysis withdrawal, and costs of acute care.

**Conclusion:**

Our model suggests nurse-led ACP leads to receipt of patient preferences for end-of-life care, but at an increased cost.

## Introduction

Advance care planning (ACP) supports people to consider and communicate their future treatment preferences in the context of their own goals and values. For people with chronic kidney disease (CKD), ACP can alleviate depression and indecision regarding the burden of dialysis, uncertainties about the future and inevitable death [1], and broaden the focus from dialysis and maintaining physical health [2, 3] to identifying and addressing goals that patients have for their remaining lives [4, 5]. ACP can also assist caregivers to overcome decisional and personal conflict and to act in accordance with patients’ end-of-life preferences [1, 6]. Yet ACP is estimated to occur with only 6–49% [7–11] of people with CKD internationally.

In past empirical studies, ACP has been associated with greater adherence to treatment preferences at end-of-life and greater incidence of patients withdrawing from dialysis in accordance with their preferences compared to controls [12, 13]. However, these findings have not been replicated in large scale, high quality randomized controlled trials [14]. Moreover, while preliminary studies suggest that ACP may reduce costs in other populations [15]; economic evaluations of ACP in CKD are scarce [16]. Economic evaluation uses comparative analysis of alternative courses of action regarding costs and consequences and thus has the potential to influence policy makers. However, the funding required to deliver a structured and effective ACP program in patients with CKD is relatively unknown.

The aim of this study was to incorporate all available evidence into a decision analysis and examine the cost-effectiveness of ACP compared to usual care for older patients with end-stage kidney disease (ESKD) managed on dialysis.

## Methods

Study reporting is based on the consolidated health economic evaluation reporting standards (CHEERS) statement and the completed checklist is reported in Supporting Information File S1 Appendix [17].

We simulated the natural history of decedents on dialysis, using hospital data, and modelled the effect of a nurse-led ACP intervention on end-of-life care preferences. We constructed a decision tree model using decision analytic software (TreeAge Pro 2017, Williamstown, USA) to compare end-of-life outcomes and hospital costs following a nurse-led ACP intervention versus no ACP intervention (usual care) in older people with ESKD managed with haemodialysis. Although alternative models of ACP are described in the literature, nurse-led interventions such as the Respecting Choices program [18] (described in detail below) have most commonly been reported in CKD [19].

### Decision tree design

Decision analysis is a systematic quantitative approach for assessing the relative value of two or more alternatives. This process includes identifying, bounding and structuring a problem using a decision tree; populating the tree with probability values for each decision and its outcome; and calculating the expected value of each decision alternative to enable quantitative comparison [20]. Most often these comparisons are in terms of costs and/or health outcomes.

The person-centred benefits or health outcomes important to this population were whether treatment preferences (i.e. such as discontinuation of dialysis) were adhered to at end-of-life (yes, no). We chose this outcome because the primary goal of ACP is to help ensure people receive treatment and care consistent with their values, goals and preferences during serious and chronic illness [21]. In addition, past research indicated ACP improves end-of-life care by enabling patients’ preferences to be determined and adhered to at end-of-life [22, 23]. The costs included hospital costs in the last 12 months of life and costs of the nurse-led ACP intervention.

#### Target population and subgroups

**Fig 1:**
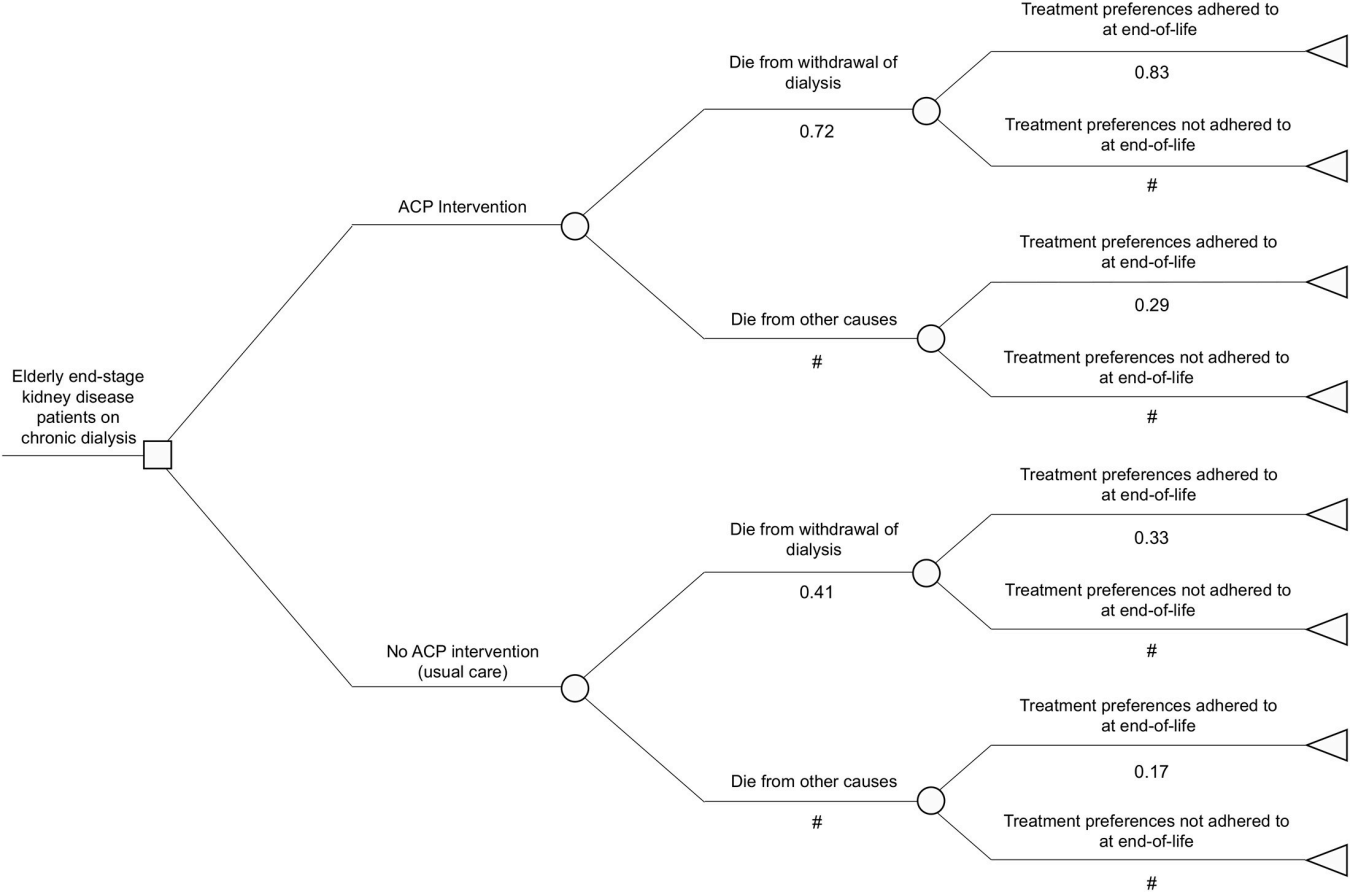
Decision tree structure of advance care planning (ACP) in older people managed with dialysis. The square symbol represents the choice of implementing ACP intervention versus no ACP intervention (usual care), the circle symbols represent the alternative chance events regarding cause of death and treatment preference being adhered to at end-of-life and the triangle symbol represents the absorbing (death) state. The hashtag symbols compliment the sum of the alternate branch probabilities to equate to 1.0.

Our model followed a hypothetical cohort of patients who were receiving dialysis; one half received the ACP intervention and the other half did not. After a period of time on dialysis, patients in both groups transitioned to one of two death states, death due to dialysis withdrawal, or death due to other causes. The final outcome was the likelihood of end-of-life treatment preferences being adhered to. We built the model to run over the last 12-months of life because this was considered the time in which the costs and effects of ACP would be incurred. The decision pathway included the probability of death due to dialysis withdrawal or death due to other causes because past studies of ACP in ESKD indicate that the probability of withdrawing from dialysis is higher for those who complete ACP compared to controls [12, 13]. Furthermore, past qualitative research has shown ACP compels some people with ESKD and their caregivers to change their beliefs and expectations about dialysis [1]. That is, some people choose to stop dialysis treatment following ACP. The structure of our decision tree is shown in Fig 1.

#### End-of-life outcomes and health system costs in hospital

We performed a comprehensive literature search to identify the best available estimates for end-of-life outcomes and health system costs following ACP in CKD. The search strategy is outlined in Supporting Information File S2 Appendix.

#### End-of-life outcomes

Probability estimates included the chance of dying from withdrawal of dialysis or dying from other causes. These estimates were sourced from studies of ACP in older people on dialysis, and from studies without an ACP intervention (representing usual care). To determine the effectiveness of ACP, probability estimates for treatment preferences being adhered to at end-of-life (yes = 1, no = 0) were identified.

We identified two studies that reported end-of-life outcomes in patients with ESKD following ACP (Table 1) [12, 13]. The study by Kirchhoff et al. (2012) included patients with both congestive heart failure and ESKD and found that patients in the intervention group were significantly more likely to have their treatment preferences adhered to at end-of-life (74% cases vs. 62% controls). In addition, patients with ESKD were significantly more likely to withdraw from dialysis in the intervention group compared to controls (38% cases vs. 17% controls).

**Table 1:** Model parameters.

Variable	Base case estimates	Low	High	Source
*Cause of death probabilities*				
Die from withdrawal of dialysis (ACP intervention)	0.72	0.30	1.0	[12, 13]
Die from withdrawal of dialysis (usual care)	0.41	0.20^†^	0.58	[3, 13]
*End-of-life probabilities*				
Die from withdrawal of dialysis and preferences adhered to at end-of-life (ACP intervention)	0.83	0.70^††^	0.99^††^	[13]
Die from withdrawal of dialysis and preferences adhered to at end-of-life (usual care)	0.33	0.15^††^	0.38^††^	[13]
Die from other causes and preferences adhered to at end-of-life (ACP intervention)	0.29	0.15^†^	0.58^†^	[13]
Die from other causes and preferences adhered to at end-of-life (usual care)	0.17	0.01^††^	0.33^††^	[13]

Abbreviations: ACP, advance care planning

^†^Estimates are for half the base case for the low end and double the base case for the high end

^††^Confidence limits for the mean

Similarly, our case control study of ACP in ESKD found that patients in the intervention group were significantly more likely to have their treatment preferences adhered to at end-of-life compared to controls (67% cases vs. 27% controls) and die from withdrawal of dialysis compared to controls (70% cases vs. 58% controls) [13]. Furthermore, we found that patients in the intervention group were significantly more likely to withdraw from dialysis in accordance with their preferences compared to controls (83% intervention vs. 33% controls).

To determine the probability of cause of death for usual care we used data reported in the 2016 Australian and New Zealand Dialysis and Transplant Association (ANZDATA) Registry [24]. The registry reported 41% of Australians aged 65 years and older died from withdrawal of dialysis.

#### Resource use and costs

Unit costs ($AUD) of care for ACP and hospital treatment received in the last 12 months were obtained from our microcosting study of ACP in ESKD at a Metropolitan hospital in Melbourne, Australia [13]. Hospital costs comprise the majority of health system costs for people on dialysis.

The health system activities involved in the nurse-led ACP intervention included: program setup, nurse and physician consultation and incidental requirements, such as recruitment and scheduling of patients, commuting to meetings and consultation with renal clinicians. A dialysis nurse was trained by attending a 2-day training workshop and by conducting eight supervised ACP conversations with an experienced clinician. ACP was introduced to patients and further meetings were scheduled with the patient and their family if the patient agreed to their family being present and if the patient wanted to proceed further with ACP discussions. Throughout the ACP intervention, supervision was provided by the ACP department Medical Director, to resolve any issues or concerns regarding the ACP consultation, to answer questions, and provide support to the ACP facilitator. In addition, the ACP facilitator attended clinical meetings in the renal unit and consulted with renal clinicians regarding patients’ treatment preferences.

During ACP consultation, the facilitator assisted patients and their family members to reflect on the patient’s goals, values and beliefs, and helped patients to consider how they would like treatment decisions to be made if they became unable to do this for themselves. In addition, the facilitator supported patients to formally appoint a substitute decision-maker, and document their preferences in an advance care directive if they wanted. If advance care directives were completed, they were stored in the medical record in the legal section, and an electronic alert was placed on the hospital system. The usual care arm did not incur any of these activities.

The main components of care in the 12-months included allied health visits, emergency department visits, intensive care unit admissions, imaging procedures, pathology tests, pharmacy services, scheduled nursing consultations (including dialysis) and surgery.

#### Model assumptions

The following model assumptions were made: First, as we identified minimal studies investigating whether end-of-life outcomes were different for people who complete a *written* advance care directive compared to people who make a *verbal* advance care plan only, end-of-life outcomes were not dependent on people in the ACP intervention completing ACP documentation. Second, the largest costs to the health system were assumed to be hospital costs (e.g. ICU admissions, dialysis and ward stays). Due to an absence of published costs for this population incurred outside of hospital these were excluded from our model.

#### Sensitivity analysis

A series of one-way sensitivity analyses were performed to evaluate the robustness of the model and to test the model parameters. Upper and lower estimates for probabilities and relative risks were obtained from the literature and, where available, 95% confidence intervals. Where plausible ranges were not available we applied a standard multiplier formula (0.5 to 2 times the point estimate) to construct upper and lower bounds.

#### Ethics

Clinical parameter estimates for the model were sourced from a case-control study (Ethics approval Austin Health’s Human Research Ethics Committee, reference number: LNR/17/Austin/555); de-identified aggregated data from an existing data registry; and other published literature.

## Results

The cost of implementing the ACP intervention was on average $519 per patient. The average cost per patient for the ACP group was $100,579 (SD = 17,356) and the proportion of patients receiving end-of-life care according to preferences was 68% (SD = 48). In the no ACP group, the average cost per patient was $87,282 (SD = 19,078) and the proportion of patients having preferences met was 24% (SD = 43). The average hospital costs incurred by patients in the last 12 months of life was higher for patients who withdrew from dialysis versus those who died from other causes ($110,696 vs. $71,737, Table 2).

**Table 2:** Summary of hospital resource use and costs for ACP and care in last 12 months of life.

		Range (AUD$)^†^	
Variable description	Base case (AU$)	High	Low
Mean per-patient ACP intervention costs			
Training for clinician	43	-	-
Consultation	75	-	-
Incidental, such as identifying and scheduling patients	326	-	-
Medical supervision	71	-	-
Total	515	1,030	258
Mean per-patient hospital costs in last 12 months of life			
Withdrawal from dialysis^‡^	110,696	221,392	55,348
Die from causes other than withdrawal from dialysis^§^	71,737	143,474	35,869

Abbreviations: ACP, advance care planning

^†^Calculation for sensitivity analyses based on multiplier formula (0.5 to 2 times the base case)

^‡^On average, costs included 195 scheduled consultations by a registered nurse or nurse practitioner;

^§^On average, costs included 166 scheduled consultations by a registered nurse or nurse practitioner

The last 12 months of life for those undergoing ACP was more expensive yet more effective in facilitating adherence to patient preferences than usual care. The incremental cost per additional case of end-of-life preferences being met (incremental cost-effectiveness ratio [ICER]) was $28,421. The results for the base case analysis are shown in Table 3. The main driver for the difference in costs and effects was that patient preferences were followed to a greater extent when dialysis was withdrawn, but that hospital costs of care were higher for those withdrawing from dialysis than those dying from other causes. For example, withdrawal from dialysis could incur 3–8 days of acute inpatient admission on the renal ward, compared with a sudden unexpected myocardial infarction that could occur in the community, with little hospital resources involved.

**Table 3:** Mean total costs per patient and effectiveness of having treatment preferences adhered to in the last 12 months of life for ACP and usual care groups.

	ACP	Usual care	Difference (95% CI)
Mean cost per patient ($AUD)	$100,579	$87,282	$13,298 ($11,697 to $14,898)
Proportion received end-of-life care according to preferences	68%	24%	44% (34% to 48%)

Abbreviations: ACP, advance care planning

### Sensitivity analysis

**Fig 2:**
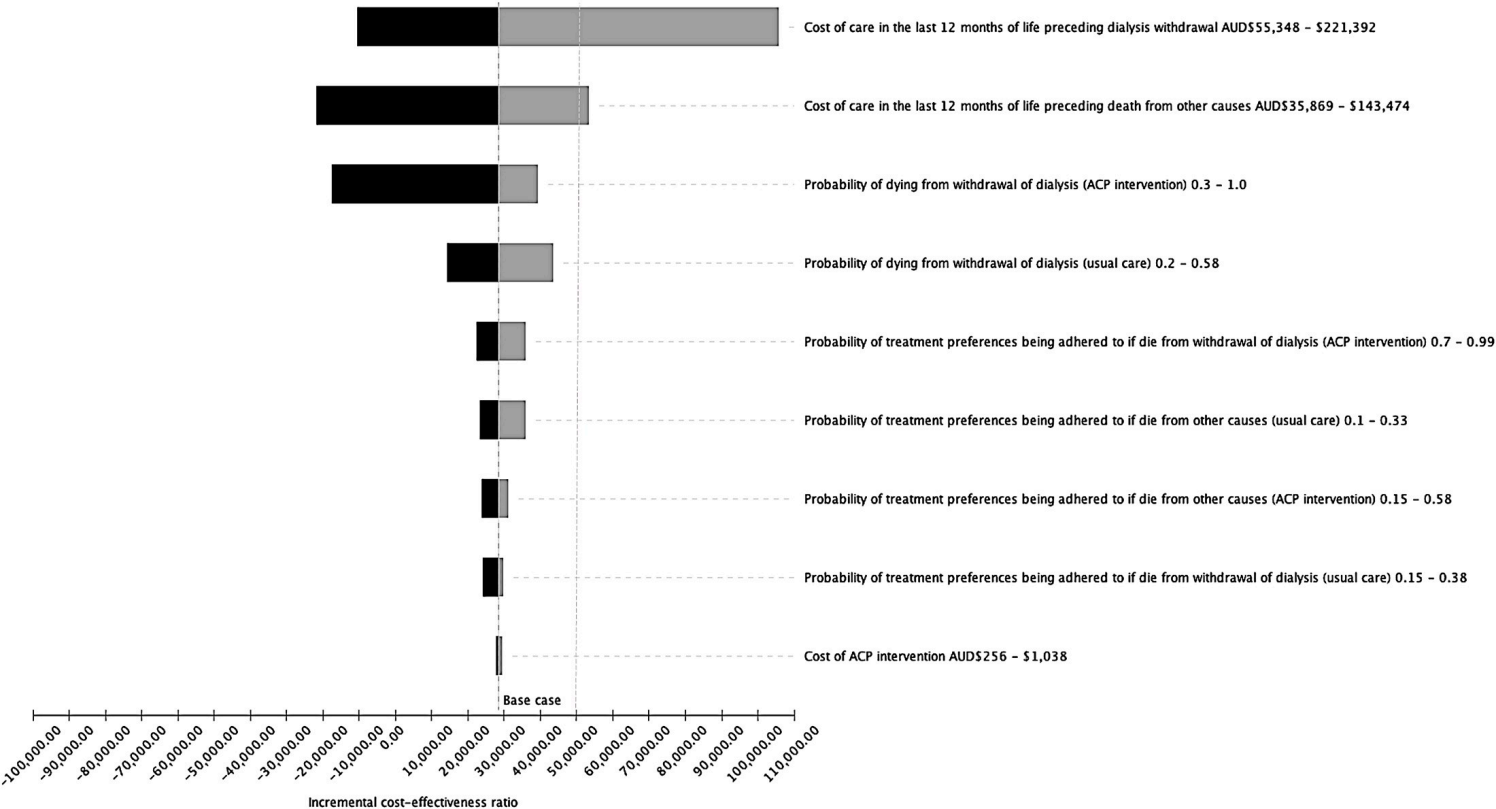
Results of one-way sensitivity analyses for ACP versus usual care. The horizontal black bars represent values for each model parameter that would lower the incremental cost-effectiveness ratio (ICER) of ACP; the grey bars represent values that would increase the ICER. For comparison, a line has been drawn at $50,000. Although this is an arbitrary threshold the Australian Government is more likely fund to fund health care interventions with an ICER of less than approximately $30,000AUD to $70,000AUD per Quality-Adjusted Life Year gained (depending on level of certainty). However, there is no known willingness to pay threshold for the outcome of this study.

The results of one-way sensitivity analyses indicated that the variables most likely to influence the ICER were the hospital costs of treatment in the last 12 months of life for those who died due to withdrawal of dialysis, costs of treatment in the last 12 months of life associated with dying from causes other than withdrawal of dialysis, and the probability of dying from withdrawal of dialysis in the intervention group. If the costs of care in the last 12-months of life preceding dialysis withdrawal were at the low end of the range ($55,348) then ACP would be cost-saving; but if the costs of care were at the high end ($221,392) then it was unlikely that ACP would be cost effective, at standard willingness to pay levels of $50,000 per unit of benefit. The cost of the ACP intervention had the least amount of influence on the ICER. These variables are presented in a tornado diagram (Fig 2) with variables of greatest influence on the cost-effectiveness results stacked at the top of the graph.

## Discussion

In our decision analysis model, ACP was more effective in facilitating adherence to treatment preferences but was more expensive than usual care for older people with ESKD managed on dialysis. Patients in the ACP group were almost three times more likely to receive end-of-life care in accordance with their preferences compared to usual care and the mean additional cost was $28,421 per patient in the last 12 months of life. The one-way sensitivity analyses showed that costs of care in the last 12 months of life (preceding withdrawal of dialysis or preceding death from other causes) and the probability of patients in the ACP group dying from withdrawal of dialysis had the greatest influence on the cost-effectiveness result. By comparison, the cost of the ACP intervention had the least influence on cost-effectiveness in the model.

Our results suggest that ACP is likely an effective intervention to facilitate adherence to the preferences of older people managed on dialysis at end-of-life. This finding is supported by one systematic review [25] and one meta-analysis [23] examining the effect of ACP on end-of-life care among differing patient groups, with both concluding that ACP increases the likelihood people will receive end-of-life care in concordance with their preferences.

In our model, for those who received ACP compared to usual care, costs were higher. This was because costs of care in the last 12 months of life preceding dialysis withdrawal were higher than costs of care for those who died from other causes. This was contrary to our expectations, as we anticipated that ACP would reduce the hospital costs of care, consistent with data from economic analysis of ACP interventions in other settings [15, 26]. Nonetheless, national studies in the US and the UK suggest that having higher rates of medical events and higher levels of morbidity are factors which influence withdrawal from dialysis [27, 28].

In our study, costs of care in the last 12-months of life were derived from a non-randomised historical control study [13], where patients who received ACP may have had higher frailty and comorbidities than patients who did not receive ACP. Therefore, hospital costs of care in the last 12-months of life may have reflected the additional care required for the management of comorbid conditions and complications of treatment rather than ACP itself.

In any case, the additional costs associated with the ACP intervention may still be good value for money depending on what society is willing to pay to facilitate adherence to end-of-life treatment preferences. The current value threshold per Quality-Adjusted Life Year (QALY) gained is $50,000 [29], which is notably higher than the mean additional cost of $28,421 per patient associated with the ACP intervention in our model. However, a similar value threshold to estimate what society is willing to pay to facilitate adherence to patient end-of-life treatment preferences in economic evaluations of ACP has not yet been described in the literature.

Achieving a sense of control and participating in treatment decisions have previously been identified by patients as components of quality end-of-life care [30, 31]. Consistent with this and a recent multidisciplinary Delphi panel which rated “care consistent with goals” as the most important patient-centred outcome rated in defining a successful ACP program [32], we undertook a novel approach to measuring cost-effectiveness in ACP and end-of-life care by focusing on the outcome of preferences being adhered to at end-of-life.

To date, QALY has been the standard outcome used in cost-effectiveness analyses in healthcare. QALY integrates both the expected number of years lived and the quality of life experienced during those years. In a cost-effectiveness analysis, the decision alternative which produces the lowest cost-per-QALY gain is recommended. However, the appropriateness of QALY in ACP and end-of-life research is debateable [27]. This is because i) the goal of ACP and end-of-life care is not necessarily to extend life, even if ACP may improve quality of life for people in the short-term and if the person does not have long left to live; and ii) QALY is almost entirely concerned with health but people with ESKD may value other outcomes arising from ACP, such as having their treatment preferences adhered to, or assisting conflicting family members to make difficult end-of-life decisions. Such factors are unaccounted for using the QALY framework and, therefore, ACP interventions maximizing life expectancy would be given priority.

Our study identified several gaps regarding the availability of person-centred data following ACP for older people with ESKD. We identified only one randomized controlled trial [12] and one historical case control study [13] that reported end-of-life outcomes for older people with ESKD following ACP. Furthermore, we did not identify any studies investigating whether end-of-life outcomes are different for people with ESKD who complete an advance care directive compared to people who make a verbal plan only during ACP. It is possible that completion of an advance care directive, in addition to having an ACP conversation only, increases the likelihood of a person’s ACP preferences adhered to at end-of-life. Lastly, although adherence to ACP preferences has previously been identified by a multidisciplinary Delphi panel as the most important person-centred metric to evaluating the quality of an ACP intervention [32]; the extent to which patients value this outcome compared to other possible outcomes at end-of-life, such as assisting conflicted caregivers, is unclear.

In our study, we used the most current evidence available and focused on the person-centred outcome of whether ACP preferences were adhered to at end-of-life. However, the study also has several limitations. Firstly, this study is not a randomized controlled trial and the data regarding costs came from a historical case control study of ACP for people with ESKD. In the only randomised controlled trial of ACP that we identified that reported end-of-life outcomes for people with ESKD, people with ESKD constituted only 42% of the sample (the rest of the sample were people with congestive heart failure). Secondly, costs of care in the last 12-months of life were estimated using one single site study and thus we acknowledge that the higher costs observed in patients who withdrew from dialysis may have been related to higher comorbidities and treatment burden rather than withdrawal of dialysis itself. Thirdly, because we found minimal data regarding the health system resources involved outside of hospital in caring for older people managed on dialysis at end-of-life, such costs were not accounted for in our model. Future studies may utilize broader, societal perspectives to conduct economic evaluations of ACP in ESKD and measure costs beyond just the perspective of the health system, such as costs to those of the patient and their family members, and at timepoints beyond the last 12-months of life. Lastly, the results may not be generalizable to non nurse-led models of ACP, such as physician or volunteer-led models.

In conclusion, our model suggests ACP leads to EOL preferences being met, and may be cost-effective depending on how much society is willing to pay to achieve this person-centred outcome. Further research is required to definitively describe the role of ACP in dialysis withdrawal, cause of death, and costs of care at end-of-life for people with ESKD managed on dialysis.

## Supporting information

S1 Appendix Consolidated health economic evaluation reporting standards—CHEERS checklist. (PDF)

S2 Appendix Search strategy. (DOCX)
