# Supplementary material for: Costs and outcomes of advance care planning and end-of-life care for older adults with end-stage kidney disease: A person-centred decision analysis
Source: PLoS One. 2019 May 31;14(5):e0217787. doi: 10.1371/journal.pone.0217787 (PMC6544277; doi:10.1371/journal.pone.0217787)
Supplement: S2 Appendix — (DOCX) [file pone.0217787.s002.docx]

**S2 Appendix. Search strategy.**

The literature search was conducted in Medline, PsycINFO, Embase, CINAHL and Google Scholar from database inception until October 2017 and through a manual search of the reference lists from relevant studies.

For cause of death and end-of-life probabilities we used the following text words or medical subject headings (MeSH):

‘advance care planning’ ‘advance directives’ plus one or more of ‘renal failure’, ‘kidney failure’, ‘end stage kidney disease’, ‘end stage renal disease’, ‘end stage kidney failure’, ‘end stage renal failure’, ‘dialysis’, ‘hemodialysis’, ‘haemodialysis’.

Randomized controlled trials, observational series, case series and population-based registry studies were all included. We excluded review articles, comments, editorials,

letters and case reports.
